# Heart of the World’s Top Ultramarathon Runner—Not Necessarily Much Different from Normal

**DOI:** 10.3390/diagnostics10020073

**Published:** 2020-01-28

**Authors:** Robert Gajda, Anna Klisiewicz, Vadym Matsibora, Dorota Piotrowska-Kownacka, Elżbieta Katarzyna Biernacka

**Affiliations:** 1 Center for Sports Cardiology at the Gajda-Med Medical Center in Pułtusk, ul. Piotra Skargi 23/29, 06-100 Pułtusk, Poland; 2 The Cardinal Stefan Wyszyński National Institute of Cardiology, ul. Alpejska 42, 04-628 Warszawa, Poland; aklisiewicz@ikard.pl (A.K.); k.biernacka@ikard.pl (E.K.B.); 3 The 2nd Department of Clinical Radiology, Medical University of Warsaw, ul. Banacha 1A, 02-097 Warsaw, Poland; vadym.matsibora@gmail.com; 4 The 1st Department of Radiology, Medical University of Warsaw, ul. Żwirki i Wigury 61, 02-091 Warsaw, Poland; dodo@mrlab.pl

**Keywords:** professional ultramarathon runner, echocardiography, electrocardiogram, magnetic resonance imaging, Cardiac ^31^P-MR spectroscopy, blood tests

## Abstract

The impact of ultramarathon (UM) runs on the organs of competitors, especially elite individuals, is poorly understood. We tested a 36-year-old UM runner before, 1–2 days after, and 10–11 days after winning a 24-h UM as a part of the Polish Championships (258.228 km). During each testing session, we performed an electrocardiogram (ECG), transthoracic echocardiography (TTE), cardiac magnetic resonance imaging (MRI), cardiac ^31^P magnetic resonance spectroscopy (^31^P MRS), and blood tests. Initially, increased cholesterol and low-density lipoprotein cholesterol (LDL-C) levels were identified. The day after the UM, increased levels of white blood cells, neutrophils, fibrinogen, alanine aminotransferase, aspartate aminotransferase, creatine kinase, C-reactive protein, and N-terminal type B natriuretic propeptide were observed. Additionally, decreases in hemoglobin, hematocrit, cholesterol, LDL-C, and hyponatremia were observed. On day 10, all measurements returned to normal levels, and cholesterol and LDL-C returned to their baseline abnormal values. ECG, TTE, MRI, and ^31^P MRS remained within the normal ranges, demonstrating physiological adaptation to exercise. The transient changes in laboratory test results were typical for the extreme efforts of the athlete and most likely reflected transient but massive striated muscle damage, liver cell damage, activation of inflammatory processes, effects on the coagulation system, exercise-associated hyponatremia, and cytoprotective or growth-regulatory effects. These results indicated that many years of intensive endurance training and numerous UMs (including the last 24-h UM) did not have a permanent adverse effect on this world-class UM runner’s body and heart. Transient post-competition anomalies in laboratory test results were typical of those commonly observed after UM efforts.

## 1. Introduction

Ultramarathon (UM) running is becoming increasingly popular [1,2]. The number of UMs organized worldwide and the number of competitors of both sexes and various ages increase every year [3,4,5,6]. Many of these UMs have a long-standing tradition and take place in different weather conditions and on various routes^1^. UMs are considered to be longer the classic marathon (i.e., >42.195 km); there is no upper limit to the distance. Regular events include 100-km and multi-week runs^2^ [7]. The longest UM in history was 5509-km, between New York and Los Angeles, and held in 1928 [8]. Currently, the longest official UM in the world that takes place regularly is the Self-Transcendence 3100-mile race, which covers a total distance of 3100 miles (4989 km)^3^. During the competition, which lasts for many days, competitors must sleep and rest between individual sections of the run, where every section is longer than the marathon (i.e., >42.195 km) [9]. Athletes train for thousands of hours over the course of years [10,11]. Training triggers physiological adaptations, including in the heart and circulatory system [12,13]. The start of the competition is associated with extreme cardiovascular efforts, and the numerous transient changes that are commonly observed in blood test results may indicate damage to the heart or a cytoprotective reaction to long-term stress (e.g., increases in troponin and N-pro brain natriuretic protein [BNP]) [14,15]. Morphological and functional changes to the heart are sometimes observed, but they are not physiological adaptations to extreme endurance efforts. These changes include atrial fibrillation [16,17], bradyarrhythmia, atrioventricular conduction block [18,19], undefined cardiac hypertrophy [20], and non-sustained ventricular tachycardia [21,22,23], which, in many cases, disappear after training is stopped [24]. Other changes, such as Phidippides cardiomyopathy or presumably exercise-induced arrhythmogenic right ventricular cardiomyopathy, pose significant threats to an athlete’s life and do not always disappear after the cessation of training [25,26]. It is not understood why some athletes develop these diseases while others who train in the same way do not. However, there is a relationship between the duration and speed of run and the amount of time spent training and the likelihood of heart disorders [27,28]. One thoroughly tested competitor with elite results who has qualified to compete among the best UM runners in the world has contributed to significantly increasing our knowledge about the potential threats to the body and heart resulting from extreme UM training and competitions. Today, this knowledge is much greater in the case of marathon runs and still scarce in the case of UM runs.

In this study, we tested this 36-year-old athlete (one of the most titled UM runners in the world) before and after the 24-h UM. We hypothesized that many years of intensive UM training as well as competing in a 24-h UM may have adverse effects on the heart and body of this world-class athlete. We assessed him using an electrocardiography (ECG), transthoracic echocardiography (TTE), magnetic resonance imaging (MRI), ^31^P magnetic resonance spectroscopy (^31^P MRS), and other laboratory tests.

## 2. Materials and Methods

### 2.1. Sports Biography and Main Achievements

The UM runner we tested was 36 years old on the day of the competition (height, 1.73 m; weight, 63 kg; body mass index, 21.05 kg/m^2^). He has a sedentary profession and works Monday to Friday from 8:00 h to 16:00 h. He has been running regularly for 20 years and has run approximately 100,000 km in his lifetime. As part of his daily training over the past year, he has run an average of 22 km/day from Monday to Friday; he runs approximately 37 km on Saturdays and Sundays. He also swims, performs gymnastics, and goes to the gym for cross-training. For many years, he has been a Polish representative in UM runs, and his performance over the past several years can be found on his official website^5^. This athlete has participated in approximately 50 UMs, the longest of which was a 48-h run (362-km crown distance, 24-h UM). He has never been injured or seriously ill. The athlete we tested is one of the most successful UM runners in the world. He is a two-time Polish Champion in the 24-h UM (2017, he ran 258.228 km during the UM) and the winner of the Spartathlon in Greece (2016, 246 km). In addition, he holds numerous other honors. In 2014, he set the Polish record for 12-h races (145.572 km). He has participated in the 24-h UM World Championships four times and has won a medal each time. In 2019, he won the 48-h race in Athens (362 km).

### 2.2. Methods

**Figure 1:**
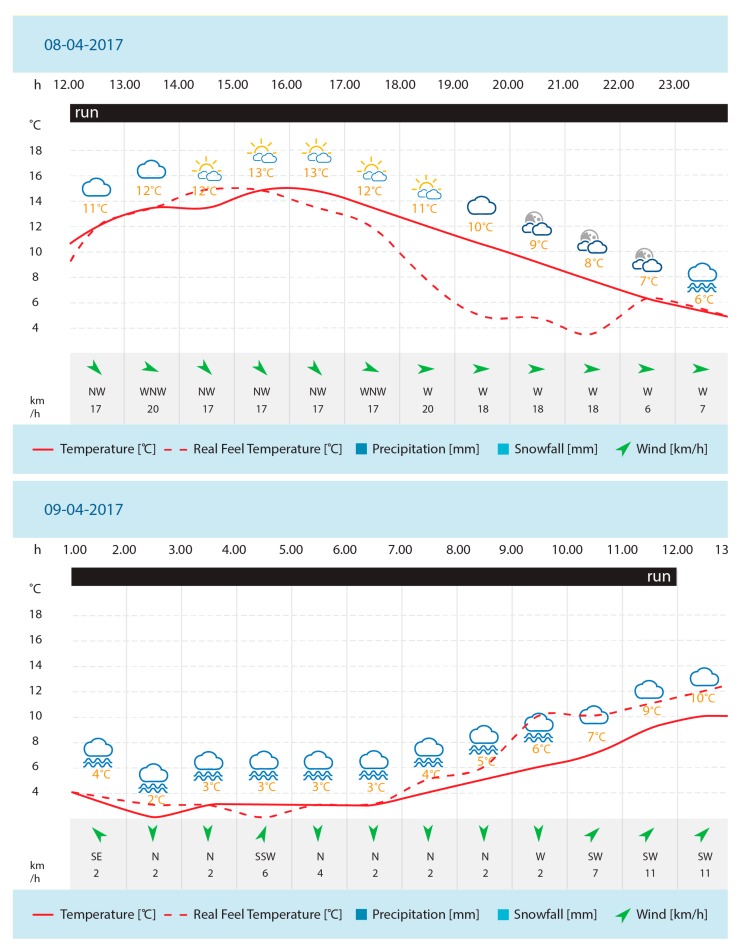
Detailed weather condition in Łódź, Poland, during a 24-h UM competition according to Global Forecast System weather (API by AccuWeather, Inc., State College, Pennsylvania, USA).

The athlete tapered his training 2 weeks before the start of the 24-h UM (i.e., gradually reduced exercise over a short period of time and then stopped completely before the competition). The run started at 12:00 h on April 8, 2017 (weather conditions are presented in Figure 1). The competitors ran on a 2-km loop (exact distance, 1.984 km) in a clockwise direction. On one of the straight sections, there was a tent in which the athlete could stop for a meal, a short rest, and basic hygiene. After 12 h, he stopped for 12–15 min for a warm meal, a change of shoes, and other hygiene activities. In addition, he used the toilet three times (2–3 min each). He ate other meals and drank liquids during the run. These competitions comprised the official Polish Championships of the 24-h UM (official website of the competition: https://pzla.pl/aktualnosci/9409-10-mistrzstwa-polski-w-biegu-24-godzinnym).

#### 2.2.1. Study Protocol

ECG, TTE, MRI, cardiac ^31^P MRS, and blood tests were performed three times. The dates and types of medical tests are presented in Table 1.

#### 2.2.2. Laboratory Examinations

Blood samples were collected from the median cubital vein with a Kima closed blood collection system. Samples were collected into the following tubes: EDTA K3 (trisodium potassium edetate) tubes for blood morphology testing; 3.2% sodium citrate tubes for coagulation tests; and clotting activator tubes for biochemical and immunochemical tests. Samples for biochemical and coagulation tests were spun in a Nuve NF 800 (Ankara, Turkey) centrifuge for 10 min at 3000 rpm. Blood morphology was analyzed on a SYSMEX XS 1000i analyzer (Kobe, Japan). Biochemical tests were conducted using serum (obtained by centrifugation) and performed using a Roche Integra 400 PLUS (Basel, Switzerland). Immunochemical tests were performed with a Roche Cobas E 411 (Basel, Switzerland). Coagulation tests were conducted using plasma and performed using a Bioksel 6000 (Grudziądz, Poland). The parameters determined were subject to internal and external laboratory controls (COBJ in DL; EQuas of Bio-Rad, Hercules, Kalifornia, USA).

#### 2.2.3. ECG Tests

Standard 12-lead ECG was performed using a Philips PageWriter TC50 apparatus (Eindhoven, Netherlands).

#### 2.2.4. Transthoracic Echocardiography

The patient underwent three complete transthoracic echocardiographic examinations (3 days before, 1 day after, and 10 days after the race) using a GE Medical System Vivid 7 (Chicago, Illinois, USA) with a 2.5-MHz transducer. M-mode, two-dimensional (2D) imaging, and Doppler techniques were used. Left ventricular (LV) end-systolic and LV end-diastolic volume and interventricular septal diastolic diameter and posterior wall thickness diameter were measured. LV systolic function was evaluated by LV ejection fraction (LVEF) and longitudinal strain (global longitudinal strain [GLS]).

LV diastolic function was evaluated using mitral inflow velocities and tissue Doppler imaging (TDI) values. The transmitral early diastolic (E-wave) velocity and atrial (A-wave) velocity were measured. The E/A ratio was calculated. Early diastolic velocity (e’) was measured in addition to E/e’ ratio.

The right ventricular end-diastolic diameter from the parasternal long-axis view and the tricuspid lateral annular systolic velocity wave (S’RV) were measured using TDI. The right atrial area (RAA) and left atrial volume index were calculated using the body surface area

#### 2.2.5. MRI

MRI was performed on a Philips Achieva 3T TX clinical scanner (Philips Medical Systems, Eindhoven, Netherlands) using the multi-coil receive mode. The protocol included B-TFE, s-TFE-GRID, Q-sFOLW SENSE images, first-pass perfusion module, and delayed enhancement images. Functions of the left and right ventricles were analyzed from short-axis cine images.

#### 2.2.6. P MRS

^31^P MRS was performed on a 1.5-T scanner (Siemens Avanto SQ T-Class, Tim (76 × 32), multinuclear option, Erlangen, Germany) at three time points: 5 days before the run, 48 h after the run, and 11 days after the run.

An ^1^H/^31^P transmit/receive heart/liver coil was used to obtain magnitude proton scout images of the heart and ^31^P spectra. ECG-gated multiple voxel ^31^P chemical shift imaging was used for cardiac ^31^P-MRS. The ratio of phosphocreatine (PCr) to adenosine triphosphate (ATP) was measured from the interventricular septum. The three spectra of the highest quality from each time point were chosen for analysis and the mean results were obtained.

#### 2.2.7. Ethical Approval

This case report was approved by the ethical review board of the Bioethics Committee of the Healthy Lifestyle Foundation in Pułtusk (EC 3/2017/medicine/sports, approval date: 30 March 2017). The runner provided his written informed consent to participate in the analysis and for his data to be published.

## 3. Results

### 3.1. Laboratory Examinations (Morphological, Biochemical, Coagulation)

Baseline results were all normal except for cholesterol and LDL-C, which were increased. On the first day after the UM, the following were increased relative to baseline: white blood cells, neutrophils, fibrinogen, alanine aminotransferase, aspartate aminotransferase, creatine kinase (CK), C-reactive protein, and N-terminal type B natriuretic propeptide. In addition, hemoglobin and hematocrit were below normal levels, and cholesterol and LDL-C levels decreased to normal values. Furthermore, the athlete had hyponatremia. On day 10, all results returned to normal and cholesterol and LDL-C returned to their original abnormal values (Table 2).

### 3.2. Electrocardiography

**Figure 2:**
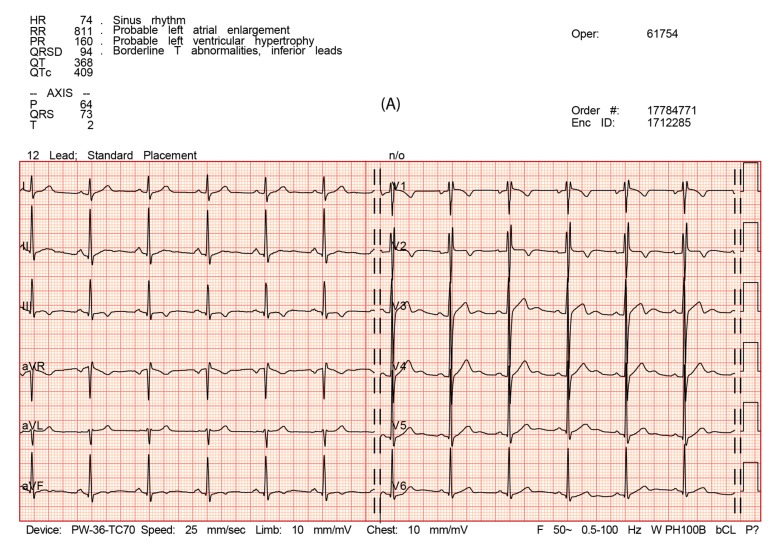

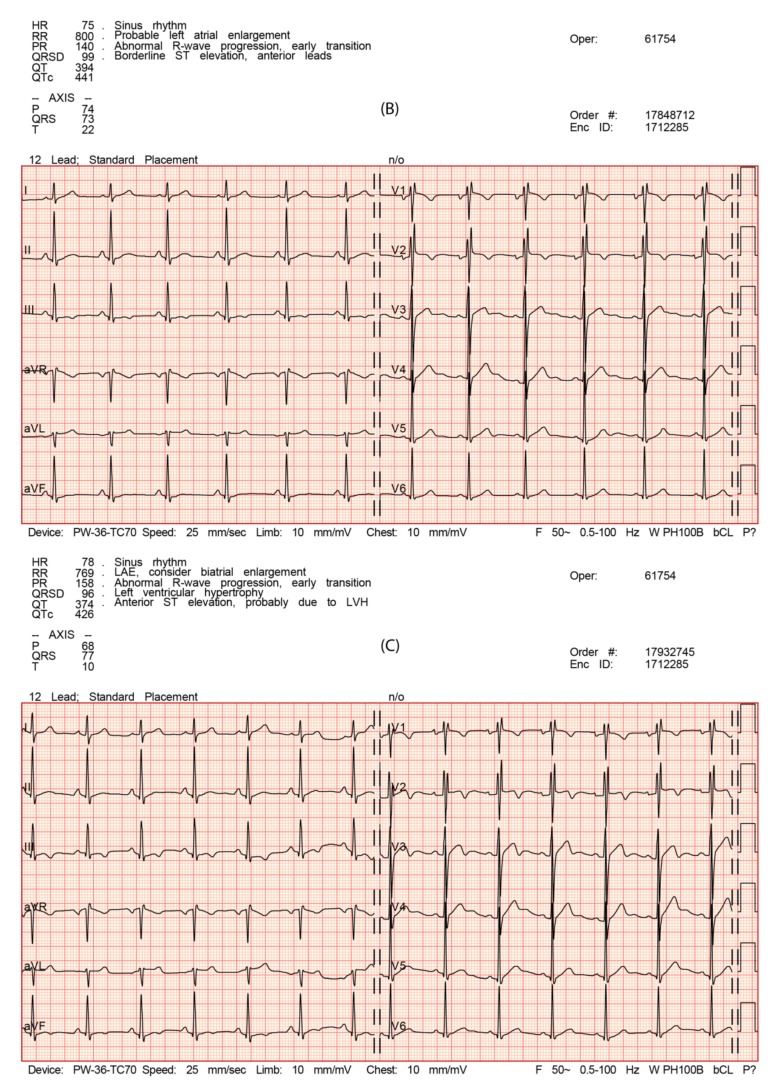
(**A**) ECG: before the run. (**B**). ECG: 1 day after the run. (**C**). ECG: 10 days after the run.

During the ECG, we observed sinus rhythm, left atrial enlargement, an incomplete right bundle block, and an increase in QRS amplitude with a normal QRS axis (the QRS voltage criteria for right ventricular hypertrophy were not fulfilled on either side) before, 1 day after, and 10 days after the 24-h UM. Abnormal negative T waves in III and aVF (flat in aVF 1 day after running) together with left atrial enlargement would suggest the need for further evaluation (Table 3, Figure 2A–C).

### 3.3. Echocardiography

One day after the 24-h UM, we observed an increase in left ventricle volume without a decrease in systolic performance or in EF or GLS assessment results. Indicators of diastolic function (E/e’ ratio, e’ wave) remained unchanged. An increase in the right ventricle size was noticed with a slight decrease in RV performance (s’RV) and an increase in RAA. All these changes returned to baseline after 10 days of recovery. Despite these differences, all the evaluated parameters remained within the normal ranges and were consistent with the physiology of physical training (Table 4).

### 3.4. MRI

We observed an increase in right ventricle volume after the run, but this was still within the normal range. No myocardial contraction disorders, regional perfusion abnormalities, and late enhancement areas in the myocardium were noted. No presence of myocardial edema on T2-weighted MRI was disclosed (Table 5).

### 3.5. P MRS

The mean PCr/ATP values were 1.42 at baseline, 1.26 at 2 days after the run, and 1.65 after 11 days of rest. All results were within normal limits for healthy subjects (2 days after exercise at the lower limit). The ratios of PCr to ATP in the interventricular septum on each day are presented in Table 6.

## 4. Discussion

We tested a 36-year-old UM runner before and twice after he won the 24-h UM as part of the Polish Championships. The aim of this study was to assess the impact of this event, as well as many years of training, on the athlete’s heart and body via selected laboratory and cardiac tests (ECG, TTE, MRI, and ^31^P MRS). Initially, only a few lipid parameters were abnormal. On the first day after the UM, we observed changes in some blood test results. On day 10, all the results returned to normal and the blood lipids returned to their original (albeit abnormal) values. Transient changes in laboratory test results are typical of extreme physical effort and represent the reaction of organs to this effort as a result of damage or adaptive mechanisms. Transient changes include massive striated muscle damage, a hepatic cell response, increased inflammation, and coagulation [29]. We found a slight degree of hemolysis (or hyperhydration), as well as minor damage to the cardiomyocytes or a cytoprotective reaction to prolonged stress. The transient minor hyponatremia observed with endurance sports was probably the result of the large amount of fluid consumed by the competitor during the run [30]. The observed ECG changes in athletes are difficult to clearly assess and require a final interpretation in combination with other diagnostic tests [31]. Suspected abnormalities and non-specific changes observed on the ECG were not confirmed by imaging tests, which are crucial in their verification. The TTE, MRI, and ^31^P MRS changes observed were still in the ranges considered normal for an athlete’s heart. The results of this study did not confirm the hypothesis that many years of endurance training and participating in UM competitions have had an adverse effect on this world-class runner.

### 4.1. How Much Physical Activity Is Too Much or Too Little?

Physical activity dosed individually as part of a healthy lifestyle has reached UM distances in some individuals [32]. Primary prevention and secondary prevention of cardiovascular diseases have established positions [33]. Research has indicated that there is a U-shaped relationship between the amount and intensity of physical activity and health [34,35]. It has been reported that light and moderate-intensity joggers have lower mortality rates than sedentary individuals, whereas the mortality rate of strenuous joggers is not statistically different from that of sedentary individuals [36]. Hypothetically, it can be presumed that excess activity is worse than inactivity. The results of research published thus far do not support this hypothesis. However, exercise-induced arrhythmogenic right ventricular cardiomyopathy and Phidippides cardiomyopathy are both associated with life-threatening ventricular arrhythmias [25,26]. The correlation between endurance sports and FA frequency has also been studied extensively [37]. Often, success in a multi-day UM is associated with resistance to sleep deprivation and ability to run without breaks at a low intensity. The athlete we tested ran at an average pace of 5:34:2 minutes per kilometer^4^. Exercise intensity is well-reflected by heart rate monitors used by many athletes. These monitors accurately determine the heart rate, thus allowing athletes and coaches to determine the intensity of effort throughout the various phases of a run [38,39,40].

### 4.2. Lipids

Nutrition is one of the most important elements of preparation for the UM. Extremely long efforts require an adequate energy supply, which is associated with a preferred diet. Ultra-endurance athletes who habitually consumed a very low-carbohydrate/high-fat diet for more than 1 year showed unique cholesterol profiles that were characterized by consistently higher plasma levels of LDL-C and high-density lipoprotein cholesterol (HDL-C). It has been reported that expanding the circulating cholesterol pool helps to meet the heightened demand for lipid transport in highly trained, keto-adapted athletes [41]. In this case study, we observed increased baseline levels of cholesterol and LDL-C, a significant reduction in both lipids immediately after exercise, and a return to baseline on day 10 after the UM. HDL-C was increased at all three timepoints and increased after exercise compared to baseline and during rest. A slightly different change in HDL levels was observed by Chen and others after a 24-h UM [42]. Family history of dyslipidemia is negative in the athlete observed in this study.

### 4.3. Hemoglobin

In this study, significant decreases in hemoglobin and hematocrit levels were detected 2 days after the run. Anemia due to mechanical hemolysis and oxidative blood cell damage during prolonged physical activity is suggested as the cause of the decreased red blood cell count after long runs [42,43]. However, this may also be associated with excessive fluid intake [44].

### 4.4. Enzymes and Echocardiography

Increased activity of enzymes released from the muscles (mainly CK) after exercise was first reported in 1958 [45]. CK levels depend on the intensity and duration of prior exercise and peak 24 h after training. Since the duration of exertion is the dominant factor, the highest enzymatic activity of circulating CK after exercise is found after UMs or triathlons [45]. In this case study, we observed a 50-fold increase in CK on the first day after the run. Alanine aminotransferase levels also increased significantly after the race.

Passaglia et al. have obtained different results than those of the current study after blood tests and TTE following a 24-h UM [46]. After the race, they observed a slight increase in troponin levels with decreased LVEF and LV hypertrophy, increased left atrial volume, and decreased E/A ratio in some runners on TTE. Increased troponin levels after the UM without subsequent recognition of permanent myocardial damage is a common phenomenon [47,48]. In the present case, troponin levels were in the normal range, with peak values observed after the run.

Until recently, results of diagnostic analyses of the long-term effects of running on the heart using cardiac biomarkers have been difficult to interpret. The increase in BNP after 100-km trials might show cytoprotective or growth-regulatory effects, but also myocardial insufficiency [49]. In contrast to cardiac patients, it is still unclear whether the appearance of or increase in cardiac biomarkers following exercise in obviously healthy athletes represents a clinically significant cardiac insult or is part of the physiological response to endurance exercise [50]. In this study, the highest BNP level after the run was still within normal limits.

Kosowski et al. reported that marathon running is associated with sharp and significant increases in biomarkers of cardiovascular stress. The profile of these changes, however, along with echocardiographic parameters, did not suggest irreversible myocardial damage [51]. Neilan et al. (2006) screened non-elite Boston Marathon participants using echocardiography and serum biomarkers [47]. Echocardiographic abnormalities after the race were only partly consistent with our results and included altered diastolic filling, increased pulmonary pressures and right ventricular dimensions, and decreased right ventricular systolic function. Unfortunately, no further observations were made.

Myocardial speckle tracking echocardiograms of the right ventricle and left ventricle were obtained before and immediately after a 161-km UM in healthy adults [52]. According to this study, RV size was significantly increased after the race, and there was an increase in LV eccentricity index. The mechanisms responsible for these findings were not clear. Scott et al. (2004) tested UM runners who completed a 160-km race [53]. In contrast to our TTE results, these authors observed post-exercise decreases in LV function after the UM. Similar to our findings, N-terminal type B natriuretic propeptide concentrations increased significantly. Furthermore, transient RV (but not LV) dysfunction during TTE after a 163-km UM at a high altitude has been observed [54]. Some athletes developed marked RV dilation and hypokinesia, paradoxic septal motion, pulmonary hypertension, and wheezing. In all but one subject, cTnI was undetectable

### 4.5. MRI

One of the largest UM studies performed using a portable MRI apparatus was conducted during a transcontinental UM (4500 km over the course of 64 days) [9]. According to Klenk et al. [55], LV mass increased significantly over the course of the race, but no other significant changes were observed. It was concluded that the observed structural cardiac change indicated a physiological adaptation to excessive cardiac volume [55]. During our MRI examination, we did not observe these changes or any other abnormalities (nor did we observe gadolinium late enhancement). However, the competition had different characteristics.

### 4.6. Cardiac ^31^P MRS

^31^P MRS is a unique non-invasive imaging modality for probing in vivo high-energy phosphate metabolism in the human heart [56,57,58]. There are few ^31^P MRS studies of athletes [59,60,61]. In our study, all results were within normal limits for healthy subjects.

**Figure 3:**
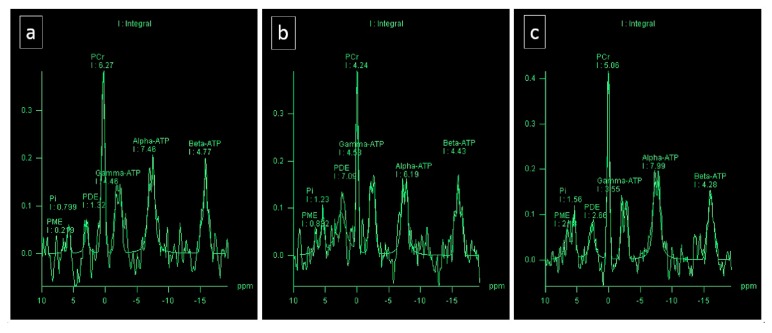
Cardiac-gated 2D CSI ^31^P spectra from the interventricular septum obtained: (**A**) before the run; (**B**) 2 days after the run; and (**C**) 11 days after the run.

PCr serves as a cellular energy reservoir; therefore, the observed reduction (average parameters) of the PCr/ATP ratio may have resulted from phosphocreatine depletion after the UM (Table 6, Figure 3). However, these were primary results based on a single-subject study and should be interpreted with caution. In the literature, normal values for the PCr/ATP ratio in healthy volunteers vary widely (range, 0.9 ± 0.3 to 2.5 ± 0.5; mean ± SD, 1.72 ± 0.26) [62,63]. Decreased PCr/ATP has been observed in many cardiac disorders such as cardiomyopathies, ischemic heart diseases, and congenital heart failure; however, significant overlap of normal and abnormal limits has been reported [57,64]. Bakermans et al. [59], investigated whether current ^31^P MRS methodology would allow for clinical applications to detect exercise-induced changes in (patho-)physiological myocardial energy metabolism and found that this was not the case. Previous studies have not allowed us to identify significant changes in cardiac metabolism during exercise [56,65].

### 4.7. What Makes Him the Champion of Ultramarathon?

The performed imaging tests did not show typical acute and chronic adaptive features found in many elite endurance athletes neither during the period of normal training nor shortly after the start of a UM. Examples of chronic features such as an eccentric LV hypertrophy in endurance athletes that reflected an increased LV internal dimension and mass, with minor changes in LV wall thickness in echocardiography, or sinus bradycardia, sinus arrhythmia, conduction delays, early repolarization of the ST segment, and isolated voltage criteria for LV hypertrophy in ECG, were not observed. Diastolic dysfunction and atrial dilation typically observed on MRI also did not occur [66]. Contrary to this and other features of Athletes’ Heart, the studied athlete has a small heart and very high EF. Even some of the typical acute post-exercise features, such as the increase in troponin levels, were not noticeable in the present case. So, what makes him a champion? 

This case study shows that a heart burdened with extreme efforts does not always respond with adaptive features described as Athletes’ Heart. It is possible that in the present case, other factors such as mental resistance, pain resistance, and endurance associated with good economics of running at a moderate pace contribute to the athlete’s success. With such a “small” heart, it is difficult to run fast. However, it is clearly still possibly be a master of the UM.

### 4.8. Strength, Limitations, and Perspectives

The main strengths of this work were its performance and ability to allow comparisons of a very wide spectrum of heart imaging results with electrocardiographic and laboratory tests from a UM runner with a history of outstanding sporting achievement. It is difficult to encourage top competitors to undergo multiple tests, especially before the start of a race. Therefore, this spectrum of research has not been performed for any UM runner to date. Undoubtedly, it is extremely rare to perform a ^31^P MRS to show the energy balance of the heart in response to a 24-h UM. The results of this study are therefore novel.

The major limitation of this study was that it only involved one subject. Another limitation was that the spectroscopic examination was performed on the second day after exercise. Due to the large amount of research performed in different locations, it was not possible to perform all tests immediately after the cessation of the effort. It would be beneficial to examine a larger number of world-class UM runners and to compare their results with those of a control group. However, with such a wide spectrum of medical tests, this would be challenging.

## 5. Conclusions

Participating in the 24-h UM after many years of professional training as a world-class UM runner did not cause permanent abnormal changes, as assessed by ECG, TTE, MRI, and ^31^P MRS. The observed changes reflected adaptation to effort. Transient anomalies in laboratory test results at 24 h after the UM were typical of those commonly observed after endurance efforts.

## Figures and Tables

**Table 1:** Date, place, and type of medical tests.

Days before or after the Run	Before Run	1 Day after the Run	2 Days after the Run	10 Days after the Run	11 Days after the Run
ECG	x	x		x	
TTE	x	x		x	
Blood tests	x	x		x	
MRI	x	x		x	
Cardiac ^31^P MRS	x		x		x

ECG, electrocardiogram; TTE, transthoracic echocardiography; MRI, magnetic resonance imaging; ^31^P MRS, cardiac ^31^P magnetic resonance spectroscopy.

**Table 2:** Summary results of blood tests.

Parameters	Units	Before Run	1 Day after the Run	10 Days after the Run	Reference Values
**Morphology**					
White blood cells	10^9^/L	4	↑ 10.87	5.36	4.0–10.0
Neutrophils	10^9^/L	↓1.81	↑ 8.46	2.67	2.5–5.0
Neutrophils (%)	%	45.2	↑ 77.8	49.8	45.0–70.0
Lymphocytes	10^9^/L	↓ 1.22	↓ 1.18	1.74	1.5–3.5
Lymphocytes (%)	%	30.5	↓ 10.9	32.5	20.0–45.0
Monocytes	10^9^/L	0.44	0.72	0.54	0.2–0.8
Monocytes (%)	%	↑ 11.0	6.6	↑10.1	3.0–8.0
Eosinophils	10^9^/L	↑ 0.46	↑0.49	0.36	0.04–0.40
Eosinophils (%)	%	↑ 11.5	4.5	↑6.7	1.0–5.0
Basophils	10^9^/L	0.07	0.02	0.05	0.020–0.100
Basophils (%)	%	↑ 1.8	0.2	0.9	0.0–1.0
Red blood cells	10^12^/L	4.96	4.39	5.02	4.1–6.2
Hemoglobin	g/dL	15	↓ 13.4	15.2	14.0–18.0
Hematocrit	%	42.8	↓ 38.0	44.2	40.0–54.0
Mean corpuscular volume	fL	86.3	86.6	88	77.0–95.0
Mean corpuscular hemoglobin concentration	g/dL	35	35.3	34.4	32.0–36.0
**Biochemistry**					
Na	mmol/L	139.28	↓ 135.8	140.02	136.0–145.0
K	mmol/L	4.75	4.13	5.12	3.8–5.2
Cl	mmol/L	99.29	98.89	101.36	98.0–110.0
Protein	g/dL	7	/	6.9	6.0–8.0
Glucose	mg/dL	↑ 105	↑109.0	95	70–99
Creatinine	mg/dL	0.76	0.74	0.64	0.30–1.20
Estimated glomerular filtration rate	mL/min/1.73 m^2^	≥60	≥60	≥60	/
Urea	mg/dL	25	35	28	<50
Uric acid	mg/dL	4.2	4.2	4.7	2.7–7.0
Alanine aminotransferase	U/L	12.74	↑73.65	32.96	<40.0
Aspartate amino transferase	U/L	18.42	↑249.18	24.93	<37.0
Gamma-G glutamyl transpeptidase	U/L	25.42	20.64	37	6.00–71.00
Amylase	U/L	51.54	47.21	56.02	10.0–100.0
Creatine kinase	U/L	102.9	↑ 5079.6	91.1	26–174
Cholesterol	mg/dL	↑ 255	177	↑ 240	115–190
HDL-C	mg/dL	67	80	72	>40
Triglycerides	mg/dL	55.04	34.9	82.81	<150.0
Low-density lipoprotein direct measured	mg/dL	185	106	163	<115
Non-HDL-C	mg/dL	188	97	168	40–160
Fe	µg/dL	120	70	134	70–181
Unsaturated iron binding capacity	ug/dL	193.9	179	197.3	150.0–349.0
Transferrin saturation	%	39.75	29.27	40.69	20.00–45.00
Total iron binding capacity	µg/dL	321.85	253.07	332.68	200–400
C-reactive protein	mg/L	0.09	↑144.12	1.52	0.0–5.0
D-Dimers	ng/mL	352.84	398.09	277.3	<113.0
N-terminal-pro hormone BNP	pg/mL	9.9	213.6	16.1	<125.00
Troponin T	pg/mL	3.67	8.11	6.32	<14
BNP	pg/mL	/	29	<2	<35
**Coagulology**					
Activated partial thromboplastin time	sek.	23.9	33	28.7	/
Activated partial thromboplastin time ratio	/	↓0.70	0.97	0.84	0.80–1.20
Prothrombin time	sek.	10.1	10.4	9.5	/
Prothrombin ratio	%	101	98	107	80.0–120.0
International normalized ratio	/	0.99	1.02	0.93	0.80–1.20
Fibrinogen	g/L	3.7	↑ 8.24	↑ 4.26	1.80–4.00

BNP, brain natriuretic protein; HDL-C, high-density lipoprotein cholesterol, ↓- under the reference values, ↑ - above the reference values.

**Table 3:** Electrocardiography parameters.

Parameters	Units	Before Run	1 Day after the Run	10 Days after the Run
Rhythm	/	Sinus, 74’	Sinus, 75’	Sinus, 78’
PQ duration	ms	160	140	160
The length of PII	ms	120	100	120
P V1 negative deflection amplitude	mm	1	1.5	1
QRS duration	ms	100	100	96
rSr’ (leads where present)	/	V1-V2	V1-V2	V1-V2
QRS morphology	/	rSr’ V1-V2	rSr’ V1-V2	rSr’ V1-V2
QRS voltage criteria for left ventricular hypertrophy SV1 + RV5 or RV6 >3.5 mV (35 mm)	mm	32/16?	32/16	32/16
QRS voltage criteria for right ventricular hypertrophy RV1 + SV5 or SV6 >1.1 mV (11 mm)	mm	5.0/1?	4.5/1	4.5/1
QTc duration	ms	410	440	420
T negative (leads when present)	/	III, aVF, V1, V2	III, V1, V2	III, aVF, V1, V2

**Table 4:** Heart systolic and diastolic function in echocardiographic parameters.

Parameters	Units (Normal Values)	Before the Run	1 Day after the Run	10 Days after the Run
Left ventricle end-diastolic diameter volume	mL (106 ± 22)	109	126	113
Left ventricle end-systolic diameter volume	mL (41 ± 10)	33	35	33
Ejection fraction 2D (%) bi-plane	% (62 ± 5)	70	72	71
Global longitudinal strain	% (−20)	20.3	21.9	20.3
Interventricular septum diameter	mm (6–10)	9	10	10
Posterior wall diastolic diameter	mm (6–10)	9	9	9
Right ventricular end-diastolic diameter	mm (20–30)	31	34	29
S’ right ventricle	cm/s (14.1 ± 2.3)	16	14	17
Left atrium	mm (30–40)	33	36	34
Left atrial volume index	mL/m^2^ (16–34)	31.8	32.3	33.5
Right atrial area	cm^2^ (16 ± 5)	17.4	20.7	18.7
Mitral valve E-wave	cm/s (73 ± 19)	87	75	75
Mitral valve A-wave	cm/s (69 ± 17)	50	57	46
E’ lateral	cm/s (>10)	21	21	20
E’ septal	cm/s (>7)	14	12	12
E/e’ lateral	ratio (<15)	4.1	3.5	3.8
E/e’ septal	ratio (<13)	6.2	6.3	6.2

**Table 5:** Magnetic resonance imaging parameters.

Parameters	Units	Before the Run	1 Day after the Run	10 Days after the Run	Reference Values
Left atrium (4CH)	cm^2^	23	24	23	15–29
LV ejection fraction	%	73	73	73	57–75
LV stroke volume	mL	102	107	104	79–135
LV end-diastolic volume index	mL/m^2^	79	83	80	66–101
LV end-systolic volume index	mL/m^2^	22	23	22	18–39
LV systolic volume index	mL/m^2^	57.6	60	58.5	43–67
LV end-diastolic volume	mL	140	148	142	121–204
LV end-systolic volume	mL	38	41	38	33–78
LV mass	g	182	182	182	109–185
Interventricular septal end diastole	mm	10	10	10	6.0–10.4
Cardiac output	L/min	7.1	7.2	7.3	2.8–8.8
Right atrium (4CH)	cm^2^	25	28	25	14–30
RV ejection fraction	%	60	63	62	50–76
RV stroke volume	mL	101	107	105	74–142
RV end-diastolic volume index	mL/m^2^	96	96.4	95.5	65–111
RV end-systolic volume index	mL/m^2^	39	36	36	18–47
RV systolic volume index	mL/m^2^	57.5	60.5	59	39–71
RV end-diastolic volume	mL	170	171	170	121–221
RV end-systolic volume	mL	69	64	65	34–94
Heart rate	bpm	70	67	70	60–90
Myocardial contraction disorders	/	no	no	no	/
Perfusion disorders	/	no	no	no	/
Delayed myocardial enhancement	/	no	no	no	/
Tricuspid valve regurgitation	/	+ mild	+ mild	+ mild	/
Myocardial edema on T2-weighted magnetic resonance imaging	/	no	no	no	/

LV, left ventricle/left ventricular; RV, right ventricle/right ventricular.

**Table 6:** The ratio of phosphocreatine (PCr) to adenosine triphosphate (ATP) in the interventricular septum on particular days.

**Parameters**	**Before Run, Middle Part, IVS**	**Before Run, Anteromedial Segments, IVS**	**Before Run, Apical Part, IVS**	**Before Run, Average Measurements**
PCr	5.22	6.27	7.34	6.27
ATP	4.22	4.46	4.89	4.38
PCr/ATP	1.24	1.4	1.5	1.43
**Parameters**	**2 Days after, Middle Part, IVS**	**2 Days after, Anteromedial Segments, IVS**	**2 Days after, Apical Part, IVS**	**2 Days after, Average Measurements**
PCr	6.4	4.24	3.99	4.87
ATP	4.42	4.53	2.58	3.84
PCr/ATP	1.45	0.94	1.54	1.26
**Parameters**	**11 Days after, Middle Part, IVS**	**11 Days after, Anteromedial Segments, IVS**	**11 Days after, Apical Part, IVS**	**11 Days after, Average Measurements**
PCr	5.06	6.44	5.02	5.49
ATP	3.55	3.36	3.05	3.32
PCr/ATP	1.43	1.91	1.64	1.65

ATP, adenosine triphosphate; IVS, interventricular septum; PCr, phosphocreatine.
